# Chemical Interactions at the Interface of Plant Root Hair Cells and Intracellular Bacteria

**DOI:** 10.3390/microorganisms9051041

**Published:** 2021-05-12

**Authors:** Xiaoqian Chang, Kathryn L. Kingsley, James F. White

**Affiliations:** Department of Plant Biology, School of Environmental and Biological Sciences, Rutgers University, New Brunswick, NJ 08901, USA; xc219@scarletmail.rutgers.edu (X.C.); kathryn.l.kingsley@gmail.com (K.L.K.)

**Keywords:** endophytes, microbe–plant interactions, nitrogen fixation, nutrient exchange trap, root hairs, plant stress tolerance

## Abstract

In this research, we conducted histochemical, inhibitor and other experiments to evaluate the chemical interactions between intracellular bacteria and plant cells. As a result of these experiments, we hypothesize two chemical interactions between bacteria and plant cells. The first chemical interaction between endophyte and plant is initiated by microbe-produced ethylene that triggers plant cells to grow, release nutrients and produce superoxide. The superoxide combines with ethylene to form products hydrogen peroxide and carbon dioxide. In the second interaction between microbe and plant the microbe responds to plant-produced superoxide by secretion of nitric oxide to neutralize superoxide. Nitric oxide and superoxide combine to form peroxynitrite that is catalyzed by carbon dioxide to form nitrate. The two chemical interactions underlie hypothesized nutrient exchanges in which plant cells provide intracellular bacteria with fixed carbon, and bacteria provide plant cells with fixed nitrogen. As a consequence of these two interactions between endophytes and plants, plants grow and acquire nutrients from endophytes, and plants acquire enhanced oxidative stress tolerance, becoming more tolerant to abiotic and biotic stresses.

## 1. Introduction

Microbes are absorbed, or otherwise enter, into plant tissues in roots and aerial parts of plants [1,2,3,4,5,6]. Nonpathogenic microbes in plant tissues are called ‘endophytes’, and these are typically bacteria and fungi [7,8,9,10,11]. Some microbes are involved in the rhizophagy cycle where microbes alternate between a free-living phase in soil where they acquire nutrients, then are attracted to root tips by root exudates (sugars, organic acids and amino acids) where microbes are taken into root cells and enter the periplasmic space, between the root cell wall and plasma membrane [12,13,14,15]. Root cells secrete superoxide onto microbes, and as a result intracellular microbe cell walls are degraded and microbes become wall-less protoplasts, termed ‘l-forms’ in bacteria and ‘mycosomes’ in fungi [14,16,17,18]. Intracellular microbial protoplasts are rapidly replicated or cloned within root cells to produce many copies through a process termed ‘blebbing’ comparable to budding seen in yeast cells [17]. Intracellular replication of l-form bacteria is controlled by membrane formation by the microbe protoplast and continuous jostling by movement of microbe protoplasts around the periphery of plant cells at a rate of approximately 10 micrometers per second by cyclosis that breaks protoplasts into smaller protoplast units [15]. Cloning of protoplasts within root cells is limited by nutrients released to microbe protoplasts in exudates and regulated by oxidative degradation by root cell reactive oxygen [19]. Generally, many more microbe protoplasts are produced within root cells than originally entered cells [14,15].

Intracellular microbes also stimulate growth of root cells by an uncertain mechanism, although microbe produced growth hormones, like auxin and cytokinin, are thought to be involved [20,21]. Root hairs, in particular, are seen to be dependent on intracellular bacteria for growth, with hairs frequently showing no elongation unless bacterial protoplasts are present within them [20,21,22]. Bacteria that are replicated in root hairs accumulate in root hair tips and trigger growth spurts in the tip of the hair, with some microbe protoplasts being ejected from tips of hairs through small pores formed in the expanding wall at the growing tip [14,15]. Once ejected from root hairs the bacteria are seen to grow and reform cell walls and flagella if they possess them [15]. In the rhizophagy cycle, plant cells are thought to extract nutrients from microbes by oxidation of their cell walls with any macro- and micronutrients contained therein, and complete oxidation of some bacteria [13,14,15,23]. Superoxide produced on root cell plasma membrane-bound NADPH oxidases adjacent to the microbe protoplasts is largely responsible for oxidative activity that extracts nutrients from microbes.

Microbes are taken into all tissues of plants, including roots, leaves and vascular tissues (e.g., phloem and xylem) depending on the plant species [24,25]. The chemical interactions of plants and endophytes in plant cells and tissues is an area where we have a dearth of information. While we possess a capacity to examine genic expression changes in plants and microbes during their interactions, we largely lack tools to directly examine chemical interactions at the interface of endophytic microbes and host cells. Over the past few years since the discovery of the rhizophagy process in plant roots [12], we have pursued experiments to illuminate the chemistry of the interactions between intracellular endophytes and plant cells [14,15,18,22,24,25,26,27,28]. In this article we describe two hypothesized interrelated chemical interactions between endophytic microbes and plant cells that stem from the direct interaction of the symbionts, maintaining the symbiosis and contributing to increased plant cell growth, enhancing host oxidative stress tolerance and nitrate transfer from endophytic microbes to plant cells. The reactants in these two equations may be considered to be components or ‘vocabulary’ of the ‘cross talk’ between endophytic bacteria and host plants. We also propose a model for intracellular microbial nitrogen fixation and transfer to root cells within root hairs.

## 2. Materials and Methods

### 2.1. Plant Materials

Creeping bluegrass (*Poa reptans*) was common to experiments described in this paper, with the exception of the rice ethylene gas experiment and the *Cynodon dactylon* root hair ejection experiment. In addition, the following seeds and seedlings were used in this study: annual bluegrass (*Poa annu*a), Bermuda grass (*Cynodon dactylon*), fescue grass (*Festuca arundinacea*), rice (*Oryza sativa*), wheat (*Tritichum aestivum*), tomato (*Solanum lycopersicum*), rape (*Brassica oleracea*), dahlia (*Dahlia pinnata*) and amaranth (*Amaranthus viridis*). Untreated seeds were obtained from commercial sources, including Harris Seeds, Inc. (Rochester, NY, USA) and The Seed Ranch (Odessa, FL, USA).

### 2.2. Experimental Conditions

Seeds were germinated on sterile 0.7% agarose (low melting temp electrophoresis grade; Sigma-Aldrich, St. Louis, MO, USA) under lab ambient conditions.

### 2.3. Histochemical Protocols

#### 2.3.1. Ethylene Staining

**Figure 1:**
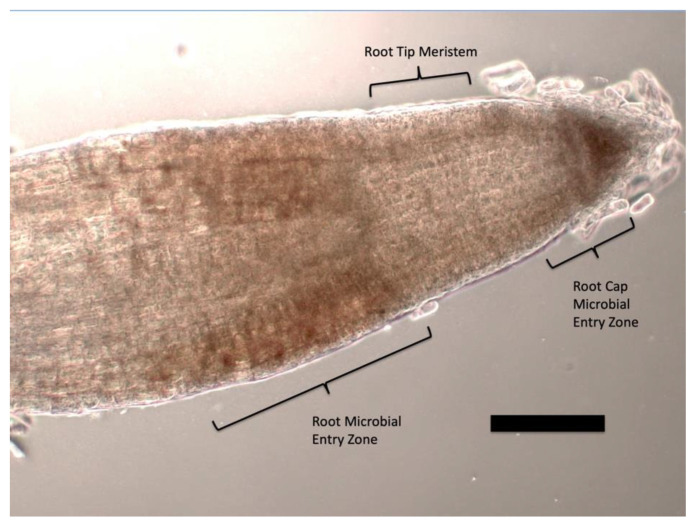
Tomato seedling root tip stained with 1% ammonium molybdate to show ethylene (purple coloration) at the ‘root microbial entry zone’ behind the root tip meristem and at the ‘root cap microbial entry zone’ at the root cap meristem (bar = 25 µm).

**Figure 2:**
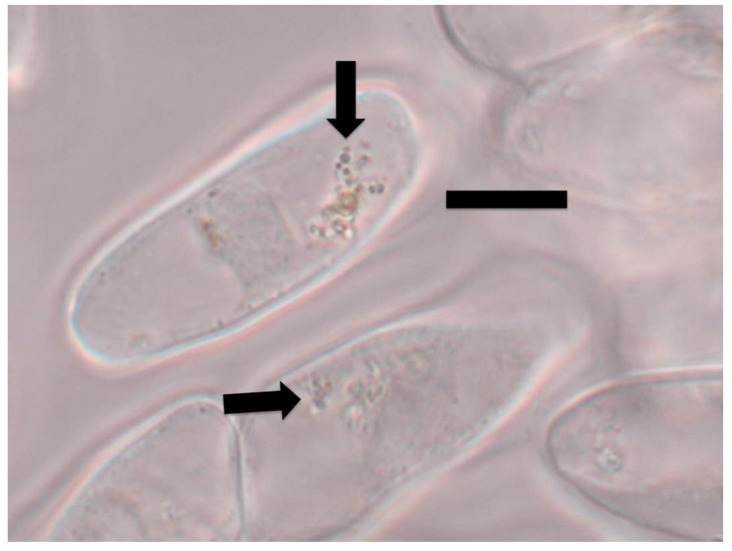
Tomato root cap cells stained with 1% ammonium molybdate to show ethylene (purple color) around bacterial protoplasts (arrow) within root cap cells (bar = 15 µm).

**Figure 3:**
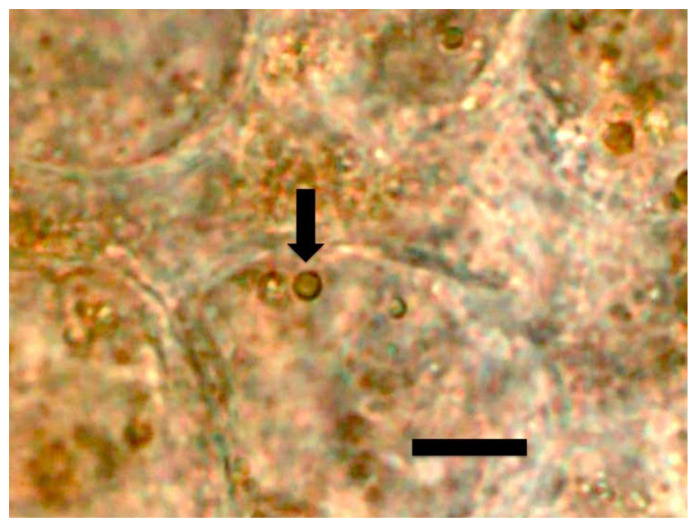
Tomato root cells at ‘root microbial entry zone’ stained with 1% ammonium molybdate to show ethylene (purple color) forming a ring around bacterial protoplasts (arrow) within root cell (bar = 5 µm).

For ethylene staining, seedlings were stained in a 1% ammonium molybdate (Sigma-Aldrich) solution in water [29] for 30 min to 2 h prior to examination using a Zeiss Axioskop compound light microscope under brightfield conditions. A blue to purple color in and around bacteria in plant root cells was a positive indication of ethylene production (Figure 1, Figure 2 and Figure 3).

#### 2.3.2. Superoxide Staining

**Figure 4:**
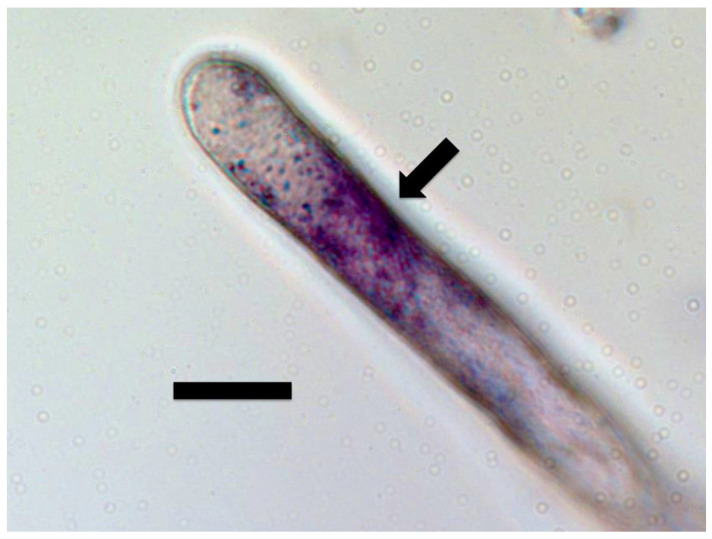
Tall fescue seedling root hair filled with bacteria and stained with 0.1% nitro blue tetrazolium showing superoxide (purple color) around bacterial protoplasts in the hair (arrow; bar = 15 µm).

**Figure 5:**
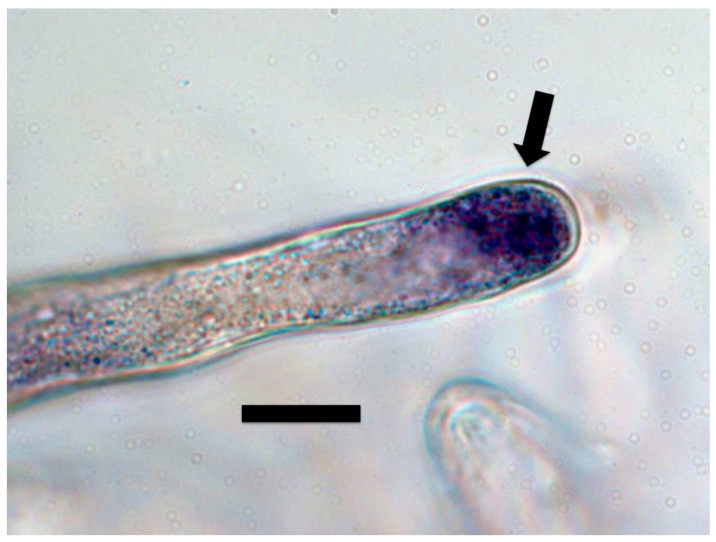
Tall fescue seedling root hair stained with 0.1% nitro blue tetrazolium showing superoxide (purple color) around bacterial protoplasts at the hair tip (arrow; bar = 15 µm).

**Figure 6:**
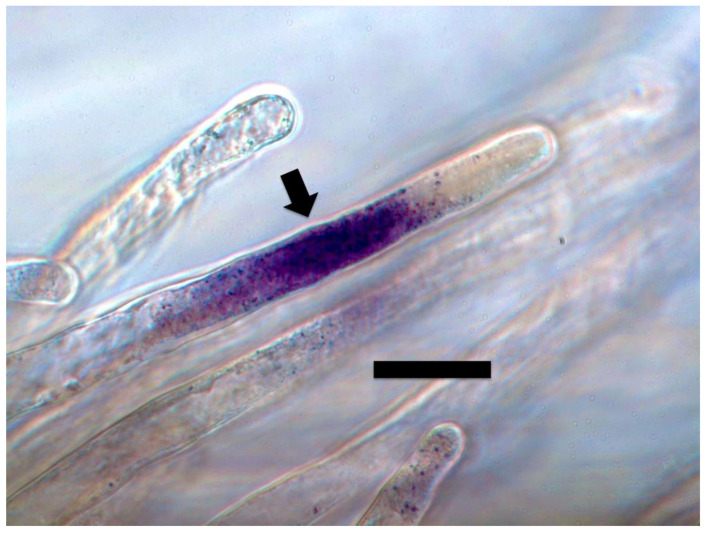
Tall fescue seedling root hair stained with 0.1% nitro blue tetrazolium showing superoxide (purple color) around bacterial protoplasts in the hair (arrow; bar = 20 µm).

To detect superoxide, a 0.1% (*w*/*v*) water solution of nitro blue tetrazolium (NBT) (Sigma-Aldrich) was used [30]. Seedling roots were soaked in NBT stain for 8–12 h at lab ambient temperature prior to examination by light microscopy as described above. Superoxide was visualized as a blue color in association with microbes (Figure 4, Figure 5 and Figure 6).

#### 2.3.3. Hydrogen Peroxide Staining

**Figure 7:**
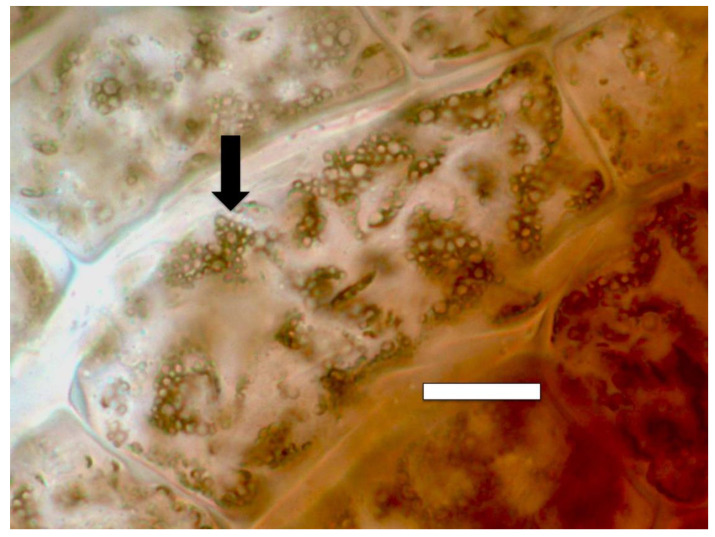
*Amaranthus viridis* seedling root cells stained with diaminobenzidine tetrahydrochloride (DAB) to show hydrogen peroxide (brown color) around clusters of bacterial protoplasts (arrow; bar = 15 µm).

**Figure 8:**
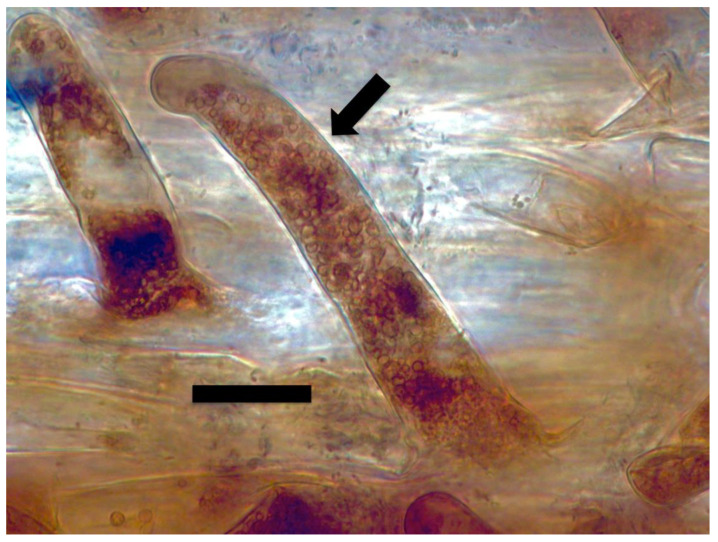
*Dahlia pinnata* seedling root hairs (arrow) stained with diaminobenzidine tetrahydrochloride (DAB) to show hydrogen peroxide (brown color) around bacterial protoplasts in hair (bar = 15 µm).

Hydrogen peroxide was visualized around microbes within root cells through use of the stain 3,3-diaminobenzidine tetrahydrochloride (DAB) [26,28]. To stain seedling roots, seedlings were immersed in the DAB solution for 5–12 h at lab ambient conditions. Hydrogen peroxide was visualized as a brown color around microbes in root cells (Figure 7 and Figure 8).

#### 2.3.4. Nitric Oxide Staining

**Figure 9:**
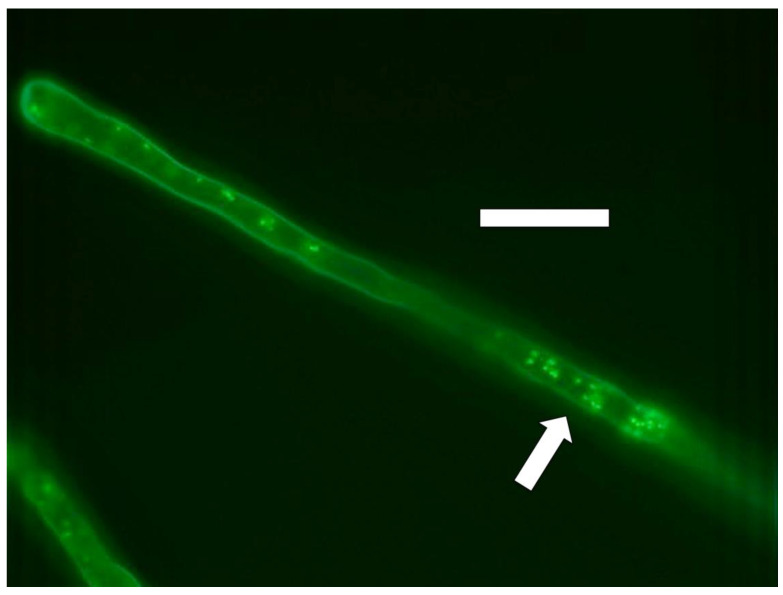
*Poa reptans* seedling root hair showing nitric oxide (green fluorescence; arrow) staining around and within cells of *Bacillus amyloliquefaciens* stained with diaminofluorescein (DAF; bar = 30 µm).

**Figure 10:**
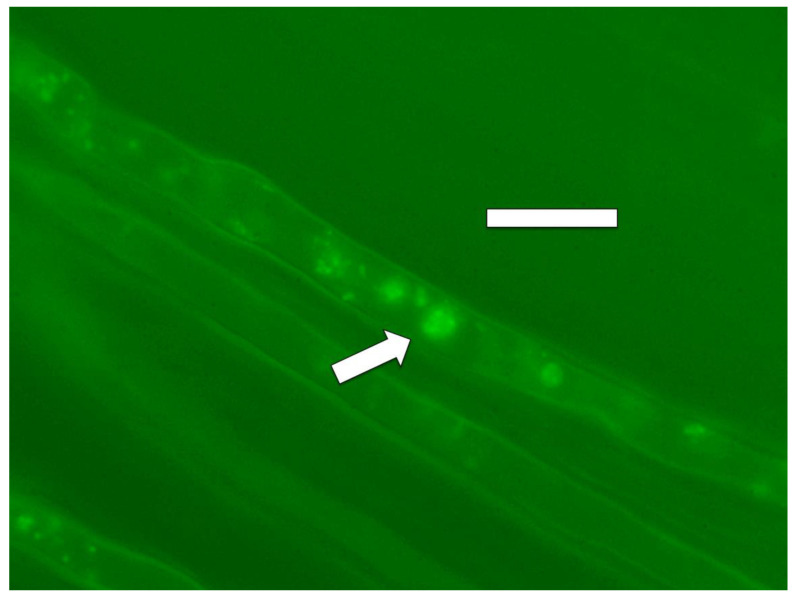
*Poa reptans* seedling root hair showing nitric oxide (green fluorescence; arrow) staining around cells of bacterium *Bacillus amyloliquefaciens* stained with diaminofluorescein (DAF; bar = 30 µm).

**Figure 11:**
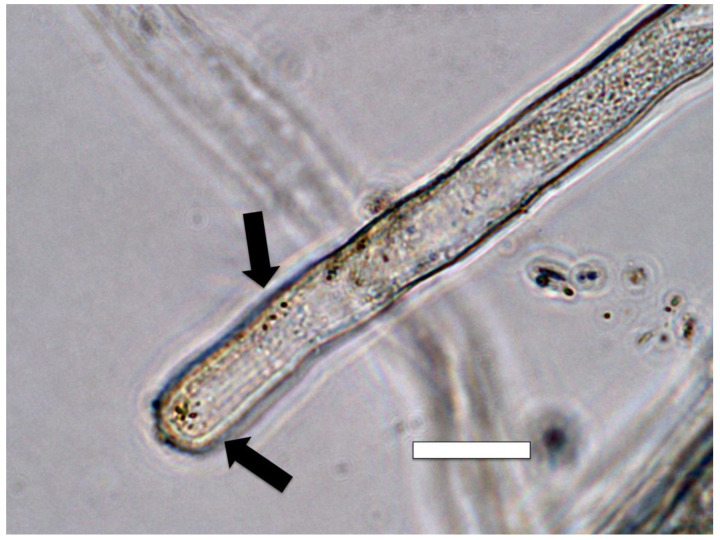
Intracellular bacteria (arrows) in *Poa reptans* root hairs stained for nitric oxide (brown color) using 0.1% iron sulfate acidified with sulfuric acid. The brown iron–nitric oxide complex is present around bacteria as a brown pigmentation (arrow) with the highest concentration very close to bacteria (bar = 15 µm).

For detection of nitric oxide around microbes in roots we employed the fluorescent indicator 4,5-diaminofluorescein (DAF) [31]. Nitric oxide was visualized in root hairs as a fluorescent haze surrounding microbes using fluorescent illumination (Figure 9 and Figure 10). We also used 0.1% ferric sulfate acidified to pH 4 with sulfuric acid to visualize nitric oxide around bacteria in root hairs [32]. Staining was accomplished by soaking roots in the stain solution for 10 min followed by microscopic examination. Nitric oxide was indicated as a brown pigmentation consisting of an iron–nitric oxide complex visualizable around bacteria within cells (Figure 11).

#### 2.3.5. Nitrate Staining

**Figure 12:**
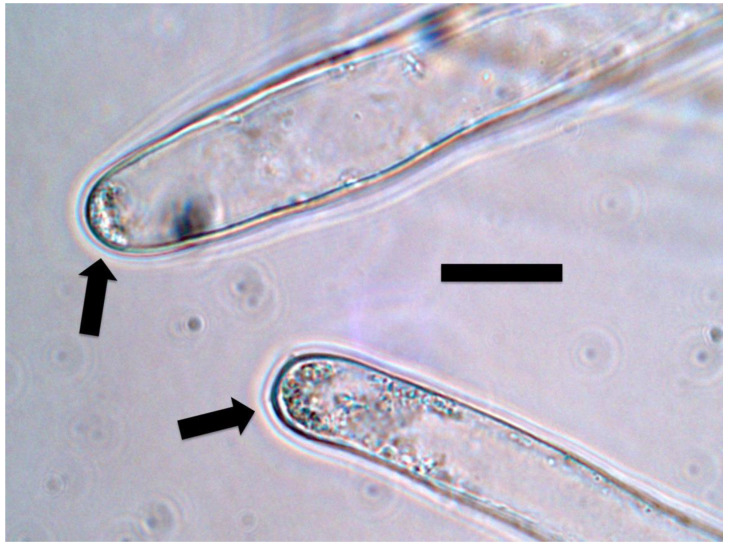
Intracellular bacteria (arrows) in oil seed rape root hair tips stained for nitrate (purple color) using 0.1% diphenylamine in 20% sulfuric acid. Nitrate is visualized around bacteria (bar = 15 µm).

**Figure 13:**
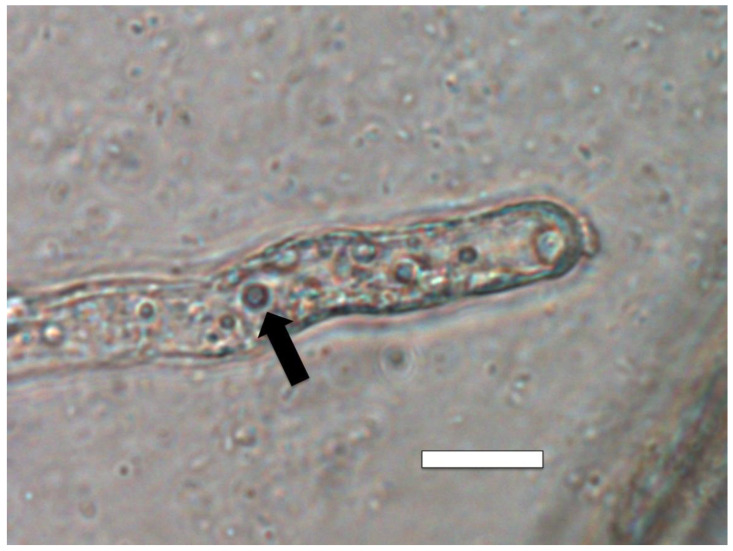
Intracellular bacteria (arrows) in *Poa reptans* root hair tips stained for nitrate (purple color) using 0.1% diphenylamine in 20% sulfuric acid. A nitrate ring (arrow) is present around bacteria in root hairs (bar = 15 µm).

Nitrate was detected around microbes in root hairs using a nitrate stain composed of 0.1% (*w*/*v*) diphenylamine (Sigma-Aldrich) in 20% (*v*/*v*) sulfuric acid [33]. Seedling roots were stained by putting excised roots on a slide with a drop of acidified diphenylamine stain. Nitrate was indicated by a blue or purple color around bacteria in root hairs (Figure 12 and Figure 13).

### 2.4. Microbe and Ethylene Experiments

#### 2.4.1. Microbe Removal and Replacement

To evaluate the effect of microbe removal from seedlings and their replacement on seeds on formation of root hairs on seedling roots, seeds of creeping bluegrass or other seeds were first surface disinfected in 4% sodium hypochlorite for 60 min to remove seed-vectored microbes. Seeds were rinsed thoroughly then placed onto agarose plates (10 plates each with 10 seeds per plate) for each seed type. Bacteria suspensions (approximately 1 × 10^6^ cells/mL) of the bacterium (*Pseudomonas fluorescens*) isolated from seeds of *Phragmites australis* were inoculated onto seeds on 5 plates for each bacterial strain. Five plates of each seed treatment were not inoculated with bacteria. After 7 days of incubation, seedlings were examined for root hair formation on seedling roots and microscopically examined to visualize presence or absence of bacteria within root cells and root hairs.

#### 2.4.2. Ethylene Gas Experiment

Seeds of rice were surface sterilized to remove bacteria, then one replicate of 3 plates (10 seeds/agarose plate) were treated by inoculating seeds with bacteria (*Pseudomonas fluorescens*), three replicates without bacteria were placed in a gas chamber with lab ambient air and three additional replicates were placed in a chamber containing 1 ppm ethylene gas. The plates were incubated under lab ambient conditions for 1 week, after which seedlings were assessed for root growth and production of root hairs.

### 2.5. Experiments with Inhibitors

#### 2.5.1. Inhibition of Superoxide

Elevated carbon dioxide gas was used to inhibit formation of superoxide by NADPH oxidases [34]. Here, seeds of *Poa annua*, *Poa reptans*, *Festuca arundinacea*, *Oryza sativa*, *Tritichum aestivum* and *Solanum lycopersicum* were planted into potting mix or agarose in magenta boxes. Magenta boxes were then placed in 2 L gas chambers (Neutec Group, Inc, Farmingdale, NY, USA). Dry ice weighing 0.6 g (measured to provide a final CO_2_ concentration of 1.3 × 10^5^ ppm in chambers) was then placed in chambers with plants and the chambers sealed. Controls were plants in chambers with air alone and sealed. Chambers were rotated daily to mix CO_2_ in air in chambers. After 7 days seedling roots were washed and examined for presence of root hairs on seedling roots. Roots were stained with 3,3-diaminobenzidine tetrahydrochloride (DAB) and counterstained with aniline blue (0.1%) to visualize microbes within root cells.

#### 2.5.2. Inhibition of Ethylene Production by Microbes

The arginine analogue *N*_ω_-Nitro-l-arginine methyl ester hydrochloride (l-NAME) (Sigma-Aldrich) is an inhibitor of microbial ethylene synthase and microbial nitric oxide synthase but does not inhibit ethylene production in plant cells [35]. We incorporated 0.5% (*w*/*v*) l-NAME into 0.7% agarose and germinated seeds of creeping bluegrass with *Pseudomonas fluorescens*. We also germinated seeds with bacteria on agarose without l-NAME. After 1 week under lab ambient conditions, we assessed seedlings for root hair growth, reactive oxygen formation and presence and condition of bacteria within root cells.

#### 2.5.3. Inhibition of Ethylene Synthesis in Plants

Aminoxyacetic acid (AOA) was used to inhibit ethylene synthesis in plants [36]. Here, AOA was incorporated into agarose at 10 µM concentration. Seedlings of creeping bluegrass were grown on this medium for 7 days. Seedlings were then assessed for root hair formation, root growth and hydrogen peroxide formation using DAB.

#### 2.5.4. Inhibition of Nitric Oxide

Methylene blue (Sigma-Aldrich) was employed as an inhibitor of nitric oxide synthase [37]. Methylene blue was incorporated into agarose at a 50 µM concentration. Seedlings were germinated and grown for 7 days then root hair growth, hydrogen peroxide formation (DAB staining) and the condition and abundance of bacteria in root hairs was assessed.

### 2.6. Cynodon dactylon Root Hair Ejection Experiment

**Figure 14:**
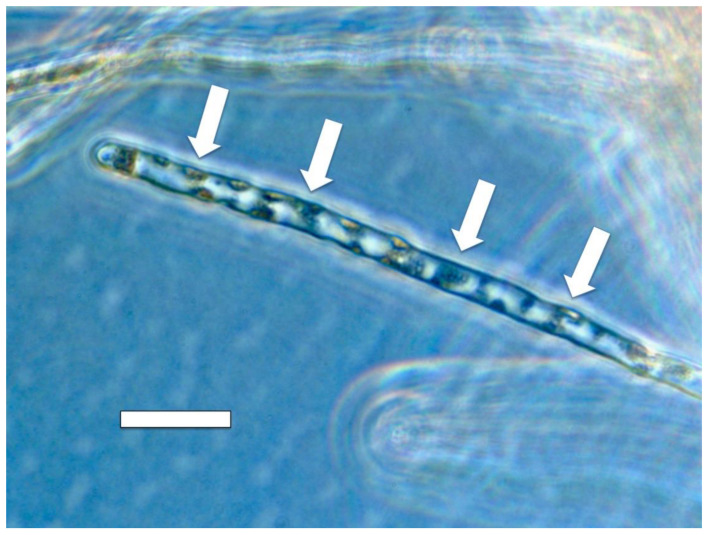
Root hair of *Cynodon dactylon* showing patches (arrows) of the bacterium *Bosea thiooxidans* (originally isolated from *Fallopia japonica*) pressed into the lateral walls of the root hair after each growth spurt of the hair (bar = 30 µm).

Pre-hulled seeds of Bermuda grass were surface sterilized by treatment with 4% NaOCl for 1 h. Seeds were rinsed with sterile water, then placed onto 0.7% agarose and inoculated with the bacterium *Bosea thioxidans* (initially isolated from Japanese knotweed (*Fallopia japonica*)). After one week, seedling roots were stained using DAB as described above and roots were examined and photographed from the reverse of Petri dishes to visualize bacteria in root hairs (Figure 14).

## 3. Results and Discussion

**Figure 15:**
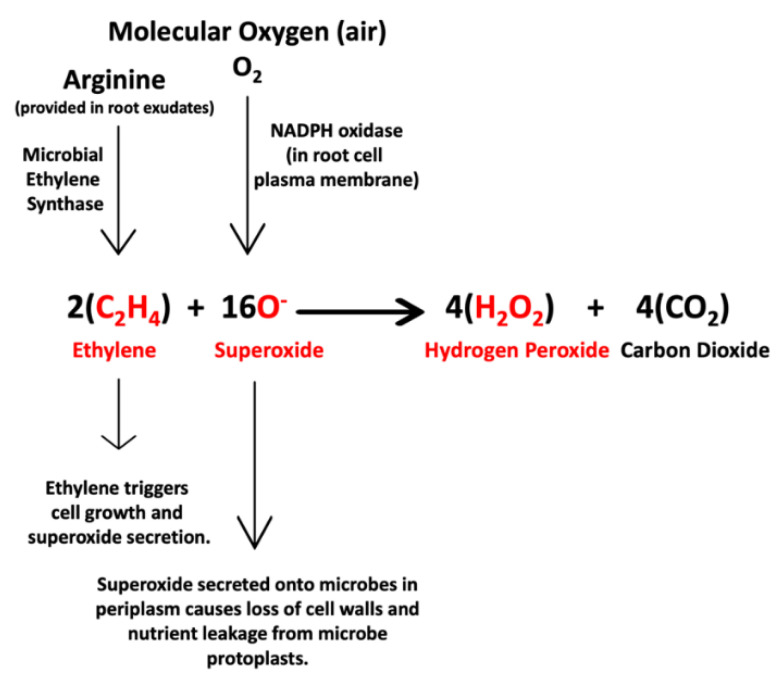
Chemical equation that represents the first interaction between endophytic bacteria and plant cells. In this interaction microbes produce and secrete ethylene onto plant cells. Ethylene triggers the plant cell to grow, release nutrients and release superoxide from NADPH oxidases (Nicotinamide Adenine Dinucleotide Phosphate Oxidases) on the plant cell plasma membrane. Some of the superoxide oxidizes cell walls from bacteria, induces nutrient leakage and may degrade them. Some of the superoxide reacts with ethylene to produce hydrogen peroxide and carbon dioxide. Reactants and products detected in histochemical analyses are indicated in red.

**Figure 16:**
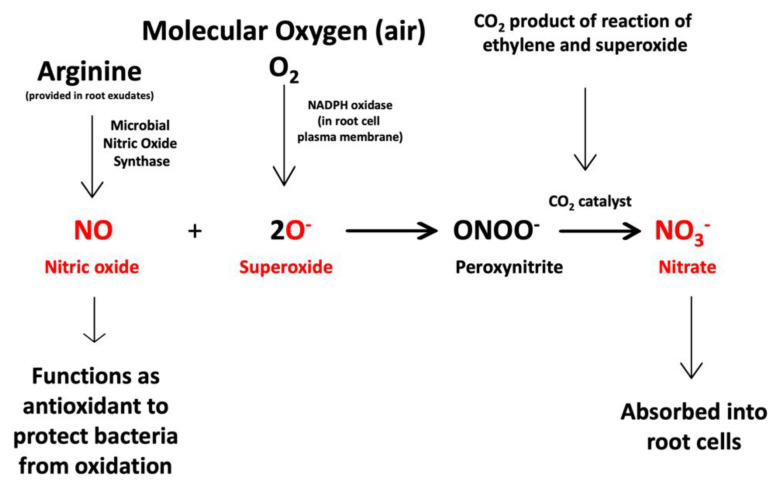
Chemical equation that represents the second interaction between endophytic bacteria and plant cells. Superoxide produced by the plant cell triggers the microbe cells to produce and secrete nitric oxide. The nitric oxide acts as an antioxidant and combines with superoxide to form short-lived peroxynitrite that is catalyzed by carbon dioxide to form nitrate. The nitrate may be absorbed into plant cells by nitrate transporters. Reactants and products detected in histochemical analyses are indicated in red.

As a result of histochemical observations and experiments that we conducted, we hypothesize that two chemical reactions (Figure 15 and Figure 16) occur at the interface between intracellular bacteria and the root cell plasma membrane.

The first equation (Figure 15) describes the first hypothesized symbiotic interaction between endophytic bacteria and plant cells. In this interaction with plants, endophytes use microbial ethylene synthase to convert arginine [38], either produced internally or supplied by host cells, to produce ethylene that is secreted into the space between microbe and host cell. The ethylene has three effects on plant cells as follows: (1) plant cell growth is initiated [39,40,41], (2) exudates containing nutrients are released from the plant cells to the microbes outside cells as plant cells grow [15], and (3) ethylene triggers superoxide production from NADPH oxidases in the plant cell plasma membrane [30,42]. Plant cell-produced superoxide has the effect of oxidizing cell walls from bacteria and inducing nutrient leakage from the bacterial protoplasts and regulating microbe proliferation and plant cell growth [9,12,14,23,25].

The second equation (Figure 16) describes the chemical response of intracellular bacterial endophytes to the superoxide produced by plant cells. The chemical response of endophytes to superoxide involves microbial secretion of nitric oxide produced from arginine by microbial nitric oxide synthase [43,44]. Nitric oxide functions as an antioxidant to protect microbes from excessive oxidative damage from superoxide [45,46,47]. The mechanism of oxidative protection of the microbes is the direct chemical reaction of nitric oxide with superoxide, where the two reactants combine to form ephemeral peroxynitrite [48]. Peroxynitrite is catalyzed by carbon dioxide to form nitrate [49,50]. This second chemical interaction is co-located in the space between intracellular bacteria and root cell plasma membrane and intertwined with the first chemical interaction because the superoxide and carbon dioxide are reactant and product of the first interaction. The nitrate product from the second chemical interaction may be absorbed directly into root cells by nitrate transporters in the membranes of root cells [51].

### 3.1. Evidence for the First Chemical Interaction

In light microscope histochemical studies, we visualized ethylene around and within microbes in root cells using 1% ammonium molybdate, where a color change from clear to blue or purple indicates presence of ethylene (Figure 1, Figure 2 and Figure 3). We visualized ethylene around microbes in root cells in the following species: *Poa reptans*, *Poa annua*, *Festuca arundinacea*, *Oryza sativa*, *Tritichum aestivum*, *Solanum lycopersicum* and *Brassica oleracea*.

Similarly, we visualized superoxide in and around microbes in numerous experiments using stain nitro blue tetrazolium (NBT) (Figure 4, Figure 5 and Figure 6). We visualized superoxide between intracellular microbes and root cells in experiments with the following species: *Brassica oleracea, Poa reptans*, *Poa annu*a, *Cynodon dactylon*, *Festuca arundinacea*, *Oryza sativa*, *Tritichum aestivum* and *Solanum lycopersicum*. Hydrogen peroxide is visualizable around bacteria within root cells using the stain 3,3-diaminobenzidine tetrahydrochloride (DAB) that results in a brown color where hydrogen peroxide is evident (Figure 7 and Figure 8). We visualized hydrogen peroxide around intracellular microbes in experiments with the following species: *Amaranthus viridis, Cynodon dactylon*, *Dahlia pinnata, Poa reptans*, *Poa annu*a, *Festuca arundinacea*, *Oryza sativa*, *Tritichum aestivum*, *Solanum lycopersicum* and *Brassica oleracea*. We did not visualize or attempt to detect the carbon dioxide product that is predicted in the reaction of ethylene and superoxide. However, experiments examining reactive oxygen and acids in root hair tips detected oscillations or pulses of reactive oxygen and acid release from growing root hair tips that correlated with growth spurts of the hairs [52]. The reactive oxygen pulses are likely due to release of superoxide and hydrogen peroxide, while acid release may be the result of CO_2_ that forms carbonic acid. It is also possible that other acids, including auxins may be released from the microbes or growing roots themselves. It has been hypothesized that auxin in the root cells that do not form hairs sustain growth that supports hair development [53].

### 3.2. Evidence for the Second Chemical Interaction

We detected nitric oxide in and around microbes within root cells using the fluorescent stain DAF (Figure 9 and Figure 10). We visualized nitric oxide around intracellular microbes in experiments with the following species: *Poa reptans*, *Poa annu*a, *Oryza sativa*, *Solanum lycopersicum* and *Brassica oleracea*. We also used acidified ferric sulfate to visualize nitric oxide around bacteria in hairs (Figure 11). We used seedlings of *Poa reptans*, *Poa annu*a and *Brassica oleracea* to visualize nitric oxide using ferric sulfate. These stains provide evidence that nitric oxide is present in root hairs in association with intracellular bacteria. An extensive literature supports possession of nitric oxide synthases by microbes [44,54], although their functions are not entirely clear [43,55,56]. There is some evidence that overproduction of nitric oxide by microbes in plants tips the balance to the microbe and leads to pathogenicity [56]. Microbe nitric oxide synthases generally use arginine to produce nitric oxide [55]. However, microbes may also produce nitric oxide by other mechanisms, including from nitrite [57]. The evidence supporting the production of superoxide was described above. Peroxynitrite is a short-lived product that is known to occur as a product of the reaction between nitric oxide and superoxide [48,50]. Peroxynitrite is a potent oxidant but it rapidly rearranges through catalysis by carbon dioxide to form nitrate. We detected nitrate in the area around intracellular microbes using the stain ‘acidified diphenylamine’ [33] where a blue or purple coloration indicates presence of nitrate (Figure 12 and Figure 13). We visualized nitrate around intracellular microbes in experiments with the following species: *Brassica oleracea*, *Festuca arundinacea*, *Poa reptans*, *Poa annu*a, *Oryza sativa* and *Solanum lycopersicum*.

### 3.3. Evidence Supporting Functions of Components of the First Chemical Interaction

Microbial ethylene triggers plant root cell growth and causes a release of nutrients (exudates) from root cells to microbes outside cells. Ethylene also triggers root cell membrane bound Nicotinamide Adenine Dinucleotide Phosphate Oxidases (NADPH oxidases) to produce superoxide from molecular oxygen. Experiments were conducted using arginine analogue l-NAME in agarose to competitively inhibit microbial ethylene synthase and formation of ethylene. In these experiments using grass seedlings (*Poa reptans*) with intracellular bacterium (*Pseudomonas fluorescens*), treatment with l-NAME resulted in seedlings in which microbes in root cells failed to produce ethylene, root cells did not produce reactive oxygen, intracellular bacteria did not lose cell walls (instead they remained in their walled rod forms), bacteria did not replicate within root cells, and as a consequence, root hairs did not elongate due to paucity of bacteria within root hair initials (trichoblasts). The sequence of events resulting in microbe protoplast formation and rapid replication within root cells by blebbing [17] and elongation of root hairs depends on an initial production of ethylene by intracellular microbes, and this elicits a response from root cells in the form of superoxide that results in degradation or control of the intracellular bacterium, essentially initiating the endophytic symbiosis. Sterilization of seeds to produce axenic seedlings also produces seedlings that do not express reactive oxygen (superoxide or hydrogen peroxide) in periplasmic spaces of root cells and on which root hair trichoblasts do not elongate [14,15]. In other experiments we used high levels of carbon dioxide gas (1.3 × 10^5^ ppm; approximately 0.3 g dry ice/L) that served as an inhibitor of NADPH oxidase, inhibiting formation of superoxide in root cells [15]. In these experiments, seedlings of the plants *Festuca arundinacea*, *Oryza sativa*, *Tritichum aestivum* and *Solanum lycopersicum* showed suppression of superoxide in the outer layers of roots. This resulted in failure of plants to oxidize cell walls from bacteria within root cells, and thus bacteria retained their cell walls; as a consequence, microbes were not replicated within root cells and root hairs did not elongate, or only short hairs formed. Normal root hair elongation requires development of a critical and sustained mass of microbe protoplasts within root hair cells. In normal root hair growth, the intracellular populations of microbes are likely not stable, but instead show reduced numbers after each hair growth spurt and ejection of microbes from tips, afterward populations of microbe protoplasts may be rebuilt in the hair tip to secrete ethylene *en masse* and trigger root hair growth again. In previous experiments [26], we treated grass seedlings with the antioxidant ascorbic acid. Seedlings treated with this antioxidant showed roots that did not produce reactive oxygen and did not produce root hairs. This experiment again shows that reactive oxygen (likely superoxide) production is necessary to facilitate the events in root hairs that result in root hair elongation. Other investigations into the molecular biology of root hair elongation have identified NADPH oxidase as critical for root hair elongation [58,59].

We further conducted an experiment using rice where we removed seed-vectored bacteria, then grew seedlings in agarose under the following conditions: (1) without bacteria in a sealed chamber containing air and 1 ppm ethylene gas, (2) with the bacterium *Pseudomonas fluorescens* in a chamber containing only air, and (3) without bacteria in a chamber containing only air. Under these conditions root hairs formed in seedlings that contained the bacterium and those without bacteria but treated with 1 ppm ethylene. No root hairs formed on seedlings without bacteria grown in air. This experiment demonstrated that microbes could be replaced by ethylene gas, supporting the function of ethylene in triggering root cell/root hair growth. These experiments are significant because in rice, ethylene, rather than auxin, appears to be the microbe-produced growth promotional hormone that largely accounts for root hair cell growth.

In another experiment, we used the plant ethylene inhibitor aminoxyacetic acid (AOA) to determine whether inhibition of plant-produced ethylene would reduce or stop root hair elongation. In this experiment, we saw an overall reduction in H_2_O_2_ secretion in roots, supporting the 1st chemical interaction, but there was no reduction in root hair elongation due to plant ethylene inhibition. This suggests that microbial ethylene alone was responsible for root hair elongation.

### 3.4. Evidence Supporting Functions of Components of the Second Chemical Interaction

Nitric oxide production in root hairs has been found to correlate with root hair elongation [60,61]. However, the precise role of nitric oxide in promotion of root hair development has not been clear [61,62]. Lombardo and Lamattina [61] showed that scavenging nitric oxide in roots resulted in anomalous development in causing shortening of root hairs and a peculiar branching with reduced vesicular inclusions in hairs, interpreted by the authors as excretory vesicles. Our experimental evidence suggests nitric oxide in root hairs is a product of intracellular bacteria and it is likely produced as an antioxidant in response to superoxide produced by root cells. In experiments where we employed the nitric oxide inhibitor methylene blue in agarose to prevent formation of nitric oxide in root hairs, root hairs showed repressed length growth, reduced numbers of microbes within hairs, and high levels of reactive oxygen staining as indicated by dark staining using the hydrogen peroxide stain DAB. In the absence of production of nitric oxide by intracellular bacteria, excess superoxide reacts with the microbes and ethylene they produce, degrading more of the intracellular microbes and reducing ethylene that is available to stimulate root hair elongation. In our experiments the lack of microbial proliferation within root hairs treated with methylene blue to suppress nitric oxide emphasizes the protective role of nitric oxide against oxidative degradation of the intracellular microbes. Nitric oxide may be an important molecule that plays a key modulation role in the symbiosis between intracellular bacteria and plant cells in being a counter to the host-produced superoxide. While the peroxynitrite product of nitric oxide and superoxide is a powerful oxidant itself, it is rapidly rearranged to nitrate through catalysis by carbon dioxide and it likely does little or no damage to microbe or plant cells.

The reaction of microbe-produced nitric oxide with plant produced superoxide to form nitrate followed by absorption by plant cells is significant in that it represents a potential mechanism whereby microbially fixed nitrogen may be transferred to plants in plants without evolved nitrogen-fixation nodules.

### 3.5. Nutrient Trap for Intracellular Microbes

**Figure 17:**
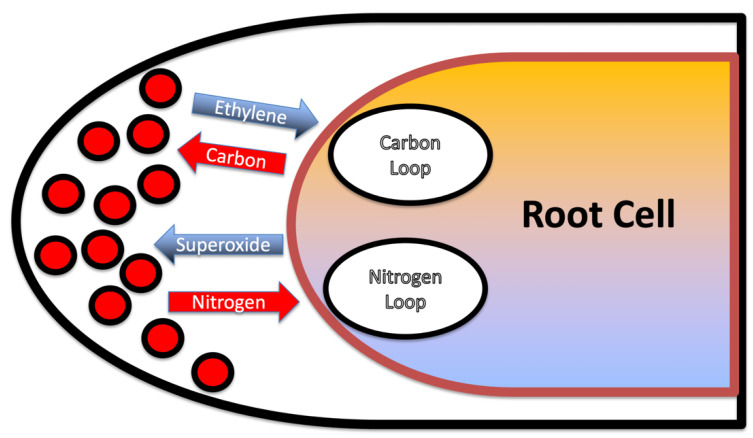
Diagrammatic representation of the nutritional loops (exchanges) that the microbes are engaged in within root cells. The carbon loop involves secretion of ethylene by microbes with a release of carbohydrate by the root cell to the microbes. The nitrogen loop involves production of superoxide by the root cell that then causes microbes to secrete nitrogen in the form of nitric oxide which is converted to nitrate and absorbed by the root cell.

The two chemical interactions that we described represent nutritional exchanges, or nutrient exchange loops, between intracellular microbes and root cells (Figure 17). The carbon loop (or photosynthate loop) involves secretion of ethylene by microbes with a release of carbohydrate by the root cell to the microbes. The nitrogen loop involves production of superoxide by the root cell that then causes microbes to secrete nitrogen in the form of nitric oxide which is converted to nitrate and absorbed by the root cell. These two nutrient exchange loops may be viewed as a trap for microbes in that microbes cannot survive within root cells if they discontinue participation in either the carbon or nitrogen nutrient exchanges. If microbes stop producing ethylene, they will not obtain photosynthate needed for growth and survival. If microbes cease secretion of nitric oxide, they will be degraded by plant produced superoxide. This nutrient trap ensures an extended engagement of microbes in nutrient exchange with the plant as long as microbes and root cells are growing.

**Figure 18:**
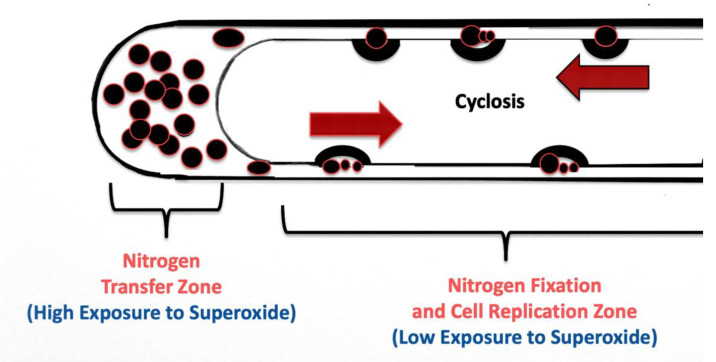
Diagrammatic representation of a root hair showing its differentiation into a nitrogen fixation and microbe replication zone along the hair length, and nitrogen transfer zone in the hair tip. Lack of movement of microbes in the tip of the hair results in a maximum exposure to superoxide and maximum secretion of nitric oxide. Rapid movement of microbes along the lateral walls reduces exposure to superoxide and replicates microbes, facilitating nitrogen fixation by the microbes.

The nutrient trap may also force microbes to acquire nitrogen, either from the host itself from secreted amino acids, or from fixation of nitrogen from the atmosphere in the soil. Under circumstances of adequate plant nitrogen, plants may provide amino acids (like arginine) in exudates to the intracellular microbes. However, in the absence of adequate plant nitrogen, plants are known to reduce secretion of amino acids in exudates [63]. This may trigger diazotrophic microbes in root hairs to fix atmospheric nitrogen in order to continue secretion of nitric oxide to counteract root cell superoxide. The cycling (cyclosis) of microbes around root hairs may be important in this nitrogen fixation (Figure 18). We have previously measured movement of microbes around the periphery of root hairs at a rate of approximately 10 µm per second [15]. This movement around root hairs is important in two ways with regard to nitrogen fixation. The constant rapid circular movement reduces exposure of diazotrophic bacteria to superoxide and facilitates the replication of the bacterial protoplasts in root cells. This creates a condition where microbe exposure to oxygen is minimized and nitrogen fixation may occur in the rapidly replicating bacterial cells. Maximum exposure to superoxide occurs at the tip of the root hair where bacterial protoplasts accumulate prior to ejection from the hair. It is likely in the tip of the root hair where microbes secrete most of the nitric oxide and where plant cells may obtain most of the nitrate. In this model root hairs show differentiation with respect to microbe nitrogen fixation (along lateral sides of hairs) and nitrogen extraction at root hair tips. If this model for nitrogen fixation in root hairs is correct, root hairs may be viewed as plant organs that facilitate nutrient exchange and facilitate nitrogen fixation and transfer to plants. The extension of root hairs into soils between soil air spaces may be for access to the oxygen for production of superoxide and nitrogen to facilitate nitrogen fixation by microbes. Absence of exposure of roots to soil air is known to cause poor nutrient absorption in many plants, and this may be due to paralysis of the nutrient extraction process in root hairs. It is notable that if correct, this nutrient exchange mechanism only functions in actively growing root hairs that only occur at the actively growing root tips. Once a root hair is fully developed all microbes have been ejected from hairs and nutrient exchange can no longer happen. Thus, this proposed nitrogen fixation mechanism exclusively occurs in actively growing roots. This is very different than nitrogen fixation in rhizobial or actinorhizal root nodules where nitrogen fixation is not tied to root cell growth and may proceed for an extended period in the nodules after roots are fully developed. In this respect, any nitrogen fixation in root hairs may be considered to be less efficient than nitrogen fixation and extraction occurring in root nodules. Additional work is needed to confirm this proposed mechanism for biological nitrogen fixation in root hairs.

### 3.6. Significance of Ethylene Secretion for Root Growth, Timing of Root Hair Growth Spurts and Ejection of Bacteria from Tips of Root Hairs

Ethylene is a growth and stress hormone in plants. The secretion of ethylene into root cells by intracellular bacteria results in increased root cell and root growth. The cyclosis driven movement of the intracellular microbes around the periphery of root cells results in an even distribution of ethylene around cells and may contribute to overall cell growth in roots, especially in the zone of cell elongation behind the root tip meristem (Figure 1). The accumulation of microbe protoplasts in the papilla of the trichoblast, or hair initial, results in high concentration of ethylene there, triggering hair elongation. Root hairs are known to grow in spurts [52]. Root hair growth spurts correlate with ejections of microbe protoplasts from the tips of hairs back into the soil [15]. The growth spurt itself is likely triggered by buildup of intracellular bacteria in the hair tip and the collective secretion of ethylene onto the hair cell at the tip, resulting in elongation of the hair. Elongation of the hair is also associated with increased reactive oxygen and decreased pH [52]. The reactive oxygen released from hair tips is likely due to release of superoxide and hydrogen peroxide, while reduced pH may be the result of carbonic acid formation as carbon dioxide is dissolved in the water in the hair tip.

**Figure 19:**
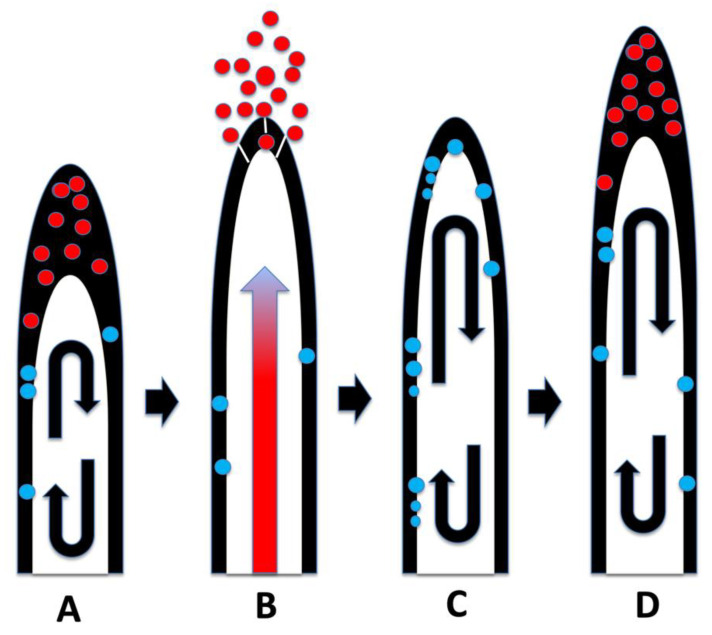
Diagrammatic representation of root hair microbe protoplast ejection. Microbes in tips are red to indicate superoxide exposure and nitrogen extraction; microbes cycling in hairs are blue to indicate replication and nitrogen fixation. (**A**) Cyclosis moves microbes to the periplasmic space at the top of the hair where they accumulate and secrete ethylene *en mass*. (**B**) Microbial ethylene triggers a growth spurt in the hair which manifests as an expansion wave that propagates from the base to the tip of the hair. The expansion wave (red arrow) causes growth in the hair and forces out the bacteria accumulated in the hair tip. (**C**) The remaining bacteria are taken back into the cyclosis stream and replicated. (**D**) As bacteria populations rebuild in root hairs, they again accumulate in the hair tips, subsequently triggering another ejection event.

Our experiments using *Cynodon dactylon* seedlings infected with the bacterium *Bosea thioxidans* grown in agarose then stained with DAB, suggest that the growth spurts and ejections of microbes occur periodically at approximately 15 min intervals (assuming a root hair growth rate of approximately 1 µm/min) [64]. In these experiments the incomplete ejection of the bacterium left clusters of bacteria adhering to the inner wall of the root hairs after each ejection event (Figure 14). Microbe ejection periodicity is likely the function of the buildup of microbes in the tips of hairs, followed by production of ethylene *en masse* that triggers growth extension in the root hair. The microbe protoplasts are ejected through pores that form in the growing root hair tip cell wall. Bacterial cell walls reform after bacterial protoplasts are ejected from hairs [14,15]. Reformation of bacterial cell walls while bacteria remain within the root hair results in failure of bacterial ejection from hair tips as seen in Figure 14. Between each ejection event the root hair takes the non-ejected microbes back into the cyclosis stream along the lateral sides of the hairs and replicates them to build up populations for their subsequent accumulation at the hair tips where the elasticity of the soft cell walls results in their accumulation there (Figure 19).

### 3.7. Increased Oxidative Stress Tolerance in Plants

Endophytes of all types have been shown to increase oxidative stress tolerance in plant hosts [27,65,66]. This increased oxidative stress tolerance is likely the result of ethylene production by endophytic microbes and the superoxide response by plant cells. The plant host must adapt to the increased stress hormone ethylene and associated reactive oxygen in its tissues by expressing stress resistance traits, including antioxidants and stress defensive features [51]. Increased oxidative stress tolerance in plants makes them more tolerant to environmental stresses (heat, soil salinity, heavy metals, diseases, etc.) that translates to increased plant hardiness. Microbe produced nitric oxide also functions to reduce stress related reactive oxygen (superoxide) formed in plants as a result of stress. In this respect, microbe produced nitric oxide may be considered a modulator of environmental stress in plants.

### 3.8. Role of Microbe-Produced Auxins in Modulating Plant Growth

The literature is replete with data regarding microbe-produced auxins and plant growth promotion, in particular with regards to root axis growth and root branching [67,68,69]. It is notable that various rhizobacteria that increase root architecture are also seen to be producers of auxins and that the amino acid precursor to auxin, tryptophane, is an abundant component of root exudates [70]. While we did not examine the presence of auxins in the interaction between intracellular microbes and plant cells, many of these microbes produce auxins in culture and may produce auxins in planta and this could contribute to plant cell growth. However, it is also possible that ethylene alone stimulates most of the growth in root hairs. Future research will be needed to examine how the suite of growth promotional molecules produced by endophytic and epiphytic microbes interact to produce growth and development in plants.

## 4. Conclusions

We found evidence for the occurrence of two chemical interactions between intracellular bacteria and root hair cells. In the first interaction, microbes produce and secrete ethylene onto plant cells. Ethylene triggers the plant cell to grow, release nutrients and release superoxide from NADPH oxidases on the plant cell plasma membrane. Some of the superoxide oxidizes cell walls from bacteria, induces nutrient leakage and may degrade them. Some of the superoxide reacts with ethylene to produce hydrogen peroxide and carbon dioxide. In the second chemical interaction, superoxide produced by the plant cell triggers microbe cells to produce and secrete nitric oxide. The nitric oxide acts as an antioxidant and combines with superoxide to form short-lived peroxynitrite that is catalyzed by carbon dioxide to form nitrate. The nitrate may be absorbed into plant cells by nitrate transporters. These two chemical interactions underlie nutrient exchanges between plant cells and intracellular bacteria. In these nutrient exchanges intracellular microbes acquire fixed carbon from plant cells, and plant cells acquire fixed nitrogen from intracellular bacteria. We do not know whether the microbial production of ethylene and nitric oxide are universal traits of endophytic microbes. Future work is needed to confirm our hypotheses regarding chemical interactions between endophytic microbes and plant cells and to evaluate our proposal regarding intracellular nitrogen fixation.

## References

[1] Doty S.L. (2017). Endophytic N-fixation: Controversy and a path forward. In Functional Importance of the Plant Microbiome.

[2] Hardoim P.R., van Overbeek L.S., Berg G., Pirttilä A.M., Compant S., Campisano A., Döring M., Sessitsch A. (2015). The hidden world within plants: Ecological and evolutionary considerations for defining functioning of microbial endophytes. Microbiol. Mol. Biol. Rev..

[3] Macedo-Raygoza G.M., Valdez-Salas B., Prado F.M., Prieto K.R., Yamaguchi L.F., Kato M.J., Canto-Canché B.B., Carrillo-Beltrán M., Di Mascio P., White J.F. (2019). Enterobacter cloacae, an endophyte that establishes a nutrient-transfer symbiosis with banana plants and protects against the black Ssgatoka pathogen. Front. Microbiol..

[4] Prieto P., Schilirò E., Maldonado-González M.M., Valderrama R., Barroso-Albarracín J.B., Mercado-Blanco J. (2011). Root hairs play a key role in the endophytic colonization of olive roots by Pseudomonas spp. with biocontrol activity. Microb. Ecol..

[5] Shehata H.R., Dumigan C., Watts S., Raizada M.N. (2017). An endophytic microbe from an unusual volcanic swamp corn seeks and inhabits root hair cells to extract rock phosphate. Sci. Rep..

[6] Thomas P., Agrawal M., Bharathkumar C.B. (2019). Diverse cellular colonizing endophytic bacteria in field shoots and in vitro cultured papaya with physiological and functional implications. Physiol. Plant.

[7] Bacon C.W., White J.F. (2016). Functions, mechanisms and regulation of endophytic and epiphytic microbial communities of plants. Symbiosis.

[8] Compant S., Duffy B., Nowak J., Clément C., Barka E.A. (2005). Use of plant growth-promoting bacteria for biocontrol of plant diseases: Principles, mechanisms of action, and future prospects. Appl. Environ. Microbiol..

[9] Foreman J., Demidchik V., Bothwell J.H.F., Mylona P., Miedema H., Angel Torres M., Linstead P., Costa S., Brownlee C., Jones J.D.G. (2013). Reactive oxygen species produced by NADPH oxidase regulate plant cell growth. Nature.

[10] Kandel S., Joubert P., Doty S. (2017). Bacterial endophyte colonization and distribution within plants. Microorganisms.

[11] Khare E., Mishra J., Arora N.K. (2018). Multifaceted Interactions between Endophytes and Plant: Developments and Prospects. Front. Microbiol..

[12] Paungfoo-Lonhienne C., Rentsch D., Robatzek S., Webb R.I., Sagulenko E., Näsholm T., Schmidt S., Lonhienne T.G.A. (2010). Turning the table: Plants consume microbes as a source of nutrients. PLoS ONE.

[13] Paungfoo-Lonhienne C., Schmidt S., Webb R.I., Lonhienne T.G.A. (2013). Rhizophagy-A New Dimension of Plant-Microbe Interactions. Molecular Microbial Ecology of the Rhizosphere.

[14] White J., Kingsley K., Verma S., Kowalski K. (2018). Rhizophagy Cycle: An Oxidative Process in Plants for Nutrient Extraction from Symbiotic Microbes. Microorganisms.

[15] White J.F., Kingsley K.L., Zhang Q., Verma R., Obi N., Dvinskikh S., Elmore M.T., Verma S.K., Gond S.K., Kowalski K.P. (2019). Review: Endophytic microbes and their potential applications in crop management. Pest Manag. Sci..

[16] Atsatt P.R., Whiteside M.D. (2014). Novel symbiotic protoplasts formed by endophytic fungi explain their hidden existence, lifestyle switching, and diversity within the plant kingdom. PLoS ONE.

[17] Errington J. (2013). L-form bacteria, cell walls and the origins of life. Open Biol..

[18] White J.F., Chen Q., Torres M.S., Mattera R., Irizarry I., Tadych M., Bergen M. (2015). Collaboration between grass seedlings and rhizobacteria to scavenge organic nitrogen in soils. Aob Plants.

[19] Kawai Y., Mercier R., Wu L.J., Domínguez-Cuevas P., Oshima T., Errington J. (2015). Cell growth of wall-free L-Form bacteria is limited by oxidative damage. Curr. Biol..

[20] Verma S.K., Kharwar R.N., White J.F. (2019). The role of seed-vectored endophytes in seedling development and establishment. Symbiosis.

[21] Verma S.K., Kingsley K., Irizarry I., Bergen M., Kharwar R.N., White J.F. (2017). Seed-vectored endophytic bacteria modulate development of rice seedlings. J. Appl. Microbiol..

[22] White J.F., Kingsley K.I., Kowalski K.P., Irizarry I., Micci A., Soares M.A., Bergen M.S. (2017). Disease protection and allelopathic interactions of seed-transmitted endophytic pseudomonads of invasive reed grass (*Phragmites australis*). Plant Soil.

[23] Hill P.W., Marsden K.A., Jones D.L. (2013). How significant to plant N nutrition is the direct consumption of soil microbes by roots?. New Phytol..

[24] White J.F., Kingsley K., Harper C.J., Verma S.K., Brindisi L., Chen Q., Chang X., Micci A., Bergen M. (2018). Reactive Oxygen Defense Against Cellular Endoparasites and the Origin of Eukaryotes. Transformative Paleobotany.

[25] White J.F., Torres M.S., Verma S.K., Elmore M.T., Kowalski K.P., Kingsley K.L. (2019). Evidence for widespread microbivory of endophytic bacteria in roots of vascular plants through oxidative degradation in root cell periplasmic spaces. PGPR Amelioration in Sustainable Agriculture.

[26] White J.F., Crawford H., Torres M.S., Mattera R., Irizarry I., Bergen M. (2012). A proposed mechanism for nitrogen acquisition by grass seedlings through oxidation of symbiotic bacteria. Symbiosis.

[27] White J.F., Torres M.S. (2010). Is plant endophyte-mediated defensive mutualism the result of oxidative stress protection?. Physiol. Plant..

[28] White J.F., Torres M.S., Somu M.P., Johnson H., Irizarry I., Chen Q., Zhang N., Walsh E., Tadych M., Bergen M. (2014). Hydrogen peroxide staining to visualize intracellular bacterial infections of seedling root cells. Microsc. Res. Tech..

[29] Lang C., Hübert T. (2012). A colour ripeness indicator for apples. Food Bioprocess Technol..

[30] Chae H.S., Lee W.S. (2001). Ethylene- and enzyme-mediated superoxide production and cell death in carrot cells grown under carbon starvation. Plant Cell Rep..

[31] Schwendemann J., Sehringer B., Noethling C., Zahradnik H.P., Schaefer W.R. (2008). Nitric oxide detection by DAF (diaminofluorescein) fluorescence in human myometrial tissue. Gynecol. Endocrinol..

[32] Holtzclaw H., Robinson W. (1988). College Chemistry with Qualitative Analysis.

[33] Coldwell B.B., McLean S.R. (1959). The reaction between diphenylamine and nitrates in ultraviolet light. Can. J. Chem..

[34] Bolevich S., Alekandr Haritonovic Kogan H., Zivkovic V., Djuric D., Aleksey Novikov A., Sergey Vorobyev I., Jakovljevic V. (2016). Protective role of carbon dioxide (CO2) in generation of reactive oxygen species. Mol. Cell. Biochem..

[35] Binder B.M., Eric Schaller G. (2017). Ethylene Signaling.

[36] Baker J.E., Anderson J.D., Adams D.O., Apelbaum A., Lieberman M. (1982). Biosynthesis of ethylene from methionine in aminoethoxyvinylglycine-resistant avocado tissue. Plant Physiol..

[37] Mayer B., Brunner F., Schmidt K. (1993). Inhibition of nitric oxide synthesis by methylene blue. Biochem. Pharm..

[38] Xia J., Yamaji N., Che J., Shen R.F., Ma J.F. (2014). Normal root elongation requires arginine produced by argininosuccinate lyase in rice. Plant J..

[39] Feng Y., Xu P., Li B., Li P., Wen X., An F., Gong Y., Xin Y., Zhu Z., Wang Y. (2017). Ethylene promotes root hair growth through coordinated EIN3/EIL1 and RHD6/RSL1 activity in Arabidopsis. Proc. Natl. Acad. Sci. USA.

[40] Lynch J., Brown K.M. (1997). Ethylene and plant responses to nutritional stress. Physiol. Plant..

[41] Pitts R.J., Cernac A., Estelle M. (1998). Auxin and ethylene promote root hair elongation inArabidopsis. Plant J..

[42] Trobacher C.P. (2009). Ethylene and programmed cell death in plants. Botany.

[43] Chaudhari S.S., Kim M., Lei S., Razvi F., Alqarzaee A.A., Hutfless E.H., Powers R., Zimmerman M.C., Fey P.D., Thomas V.C. (2017). Nitrite derived from endogenous bacterial nitric oxide synthase activity promotes aerobic respiration. MBio.

[44] Crane B.R., Sudhamsu J., Patel B.A. (2010). Bacterial nitric oxide synthases. Annu. Rev. Biochem..

[45] He H., Oo T.L., Huang W., He L.F., Gu M. (2019). Nitric oxide acts as an antioxidant and inhibits programmed cell death induced by aluminum in the root tips of peanut (*Arachis hypogaea* L.). Sci. Rep..

[46] Hummel S.G., Fischer A.J., Martin S.M., Schafer F.Q., Buettner G.R. (2006). Nitric oxide as a cellular antioxidant: A little goes a long way. Free Radic. Biol. Med..

[47] Wink D.A., Miranda K.M., Espey M.G., Pluta R.M., Hewett S.J., Colton C., Vitek M., Feelisch M., Grisham M.B. (2001). Mechanisms of the antioxidant effects of nitric oxide. Antioxid. Redox Signal..

[48] Hayashi Y., Sawa Y., Nishimura M., Fukuyama N., Ichikawa H., Ohtake S., Nakazawa H., Matsuda H. (2004). Peroxynitrite, a product between nitric oxide and superoxide anion, plays a cytotoxic role in the development of post-bypass systemic inflammatory response. Eur. J. Cardio Thorac. Surg..

[49] Meli R., Nauser T., Latal P., Koppenol W.H. (2002). Reaction of peroxynitrite with carbon dioxide: Intermediates and determination of the yield of CO_3_•− and NO_2_•. J. Biol. Inorg. Chem..

[50] Pryor W.A., Lemercier J.N., Zhang H., Uppu R.M., Squadrito G.L. (1997). The catalytic role of carbon dioxide in the decomposition of peroxynitrite. Free Radic. Biol. Med..

[51] Irizarry I., White J.F. (2018). Bacillus amyloliquefaciens alters gene expression, ROS production and lignin synthesis in cotton seedling roots. J. Appl. Microbiol..

[52] Monshausen G.B., Bibikova T.N., Messerli M.A., Shi C., Gilroy S. (2007). Oscillations in extracellular pH and reactive oxygen species modulate tip growth of Arabidopsis root hairs. Proc. Natl. Acad. Sci. USA.

[53] Jones A.R., Kramer E.M., Knox K., Swarup R., Bennett M.J., Lazarus C.M., Leyser H.M.O., Grierson C.S. (2009). Auxin transport through non-hair cells sustains root-hair development. Nat. Cell Biol..

[54] Sudhamsu J., Crane R.B. (2019). Bacterial nitric oxide synthases: What are they good for?. Trends Microbiol..

[55] Hutfless E.H., Chaudhari S.S., Thomas V.C. (2018). Emerging roles of nitric oxide synthase in bacterial physiology. Advances in Microbial Physiology.

[56] Santana M.M., Gonzalez J.M., Cruz C. (2017). Nitric oxide accumulation: The evolutionary trigger for phytopathogenesis. Front. Microbiol..

[57] Yarullina D.R., Il’inskaya O.N., Aganov A.V., Silkin N.I., Zverev D.G. (2006). Alternative pathways of nitric oxide formation in Lactobacilli: Evidence for nitric oxide synthase activity by EPR. Microbiology.

[58] Jones M.A., Raymond M.J., Yang Z., Smirnoff N. (2007). NADPH oxidase-dependent reactive oxygen species formation required for root hair growth depends on ROP GTPase. J. Exp. Bot..

[59] Wang S.S., Zhu X.N., Lin J.X., Zheng W.J., Zhang B.T., Zhou J.Q., Ni J., Pan Z.C., Zhu S.H., Ding W.N. (2018). OsNOX3, encoding a NADPH oxidase, regulates root hair initiation and elongation in rice. Biol. Plant..

[60] Lombardo M.C., Graziano M., Polacco J.C., Lamattina L. (2006). Nitric oxide functions as a positive regulator of root hair development. Plant Signal. Behav..

[61] Lombardo M.C., Lamattina L. (2012). Nitric oxide is essential for vesicle formation and trafficking in Arabidopsis root hair growth. J. Exp. Bot..

[62] Liu M., Zhang H., Fang X., Zhang Y., Jin C. (2018). Auxin acts downstream of ethylene and nitric oxide to regulate magnesium deficiency-induced root hair development in *Arabidopsis thaliana*. Plant Cell Physiol..

[63] Carvalhais L., Dennis P., Fedoseyenko D., Hajirezaei M.-R., Borriss R., von Wiren N. (2010). Root exudation of sugars, amino acids, and organic acids by maize as affected by nitrogen, phosphorus, potassium, and iron deficiency. J. Plant Nutr. Soil Sci..

[64] Grierson C., Schiefelbein J. (2002). Root hairs. Arab. Book.

[65] Mendarte-Alquisira C., Gutiérrez-Rojas M., González-Márquez H., Volke-Sepúlveda T. (2017). Improved growth and control of oxidative stress in plants of *Festuca arundinacea* exposed to hydrocarbons by the endophytic fungus *Lewia* sp.. Plant Soil.

[66] Verma S.K., Sahu P.K., Kumar K., Pal G., Gond S.K., Kharwar R.N., White J.F. (2021). Endophyte roles in nutrient acquisition, root system architecture development and oxidative stress tolerance. J. Appl. Microbiol..

[67] López-Bucio J., Campos-Cuevas J.C., Hernández-Calderón E., Velásquez-Becerra C., Farías-Rodríguez R., Macías-Rodríguez L.I., Valencia-Cantero E. (2007). Bacillus megaterium rhizobacteria promote growth and alter root-system architecture through an auxin- and ethylene-independent signaling mechanism in Arabidopsis thaliana. Mol. Plant Microbe Interact..

[68] Verbon E.H., Liberman L.M. (2016). Beneficial microbes affect endogenous mechanisms controlling root development. Trends Plant Sci..

[69] Xu L., Wu C., Oelmüller R., Zhang W. (2018). Role of Phytohormones in *Piriformospora indica*-Induced Growth Promotion and Stress Tolerance in Plants: More Questions Than Answers. Front. Microbiol..

[70] Hu L., Robert C.A.M., Cadot S., Zhang X., Ye M., Li B., Manzo D., Chervet N., Steinger T., Van Der Heijden M.G.A. (2018). Root exudate metabolites drive plant-soil feedbacks on growth and defense by shaping the rhizosphere microbiota. Nat. Commun..

